# Supplementary material for: Dispersion/dilution enhances phytoplankton blooms in low-nutrient waters
Source: Nat Commun. 2017 Mar 31;8:14868. doi: 10.1038/ncomms14868 (PMC5380962; doi:10.1038/ncomms14868)
Supplement: Supplementary Information — Supplementary Figures and Supplementary Table [file ncomms14868-s1.pdf]

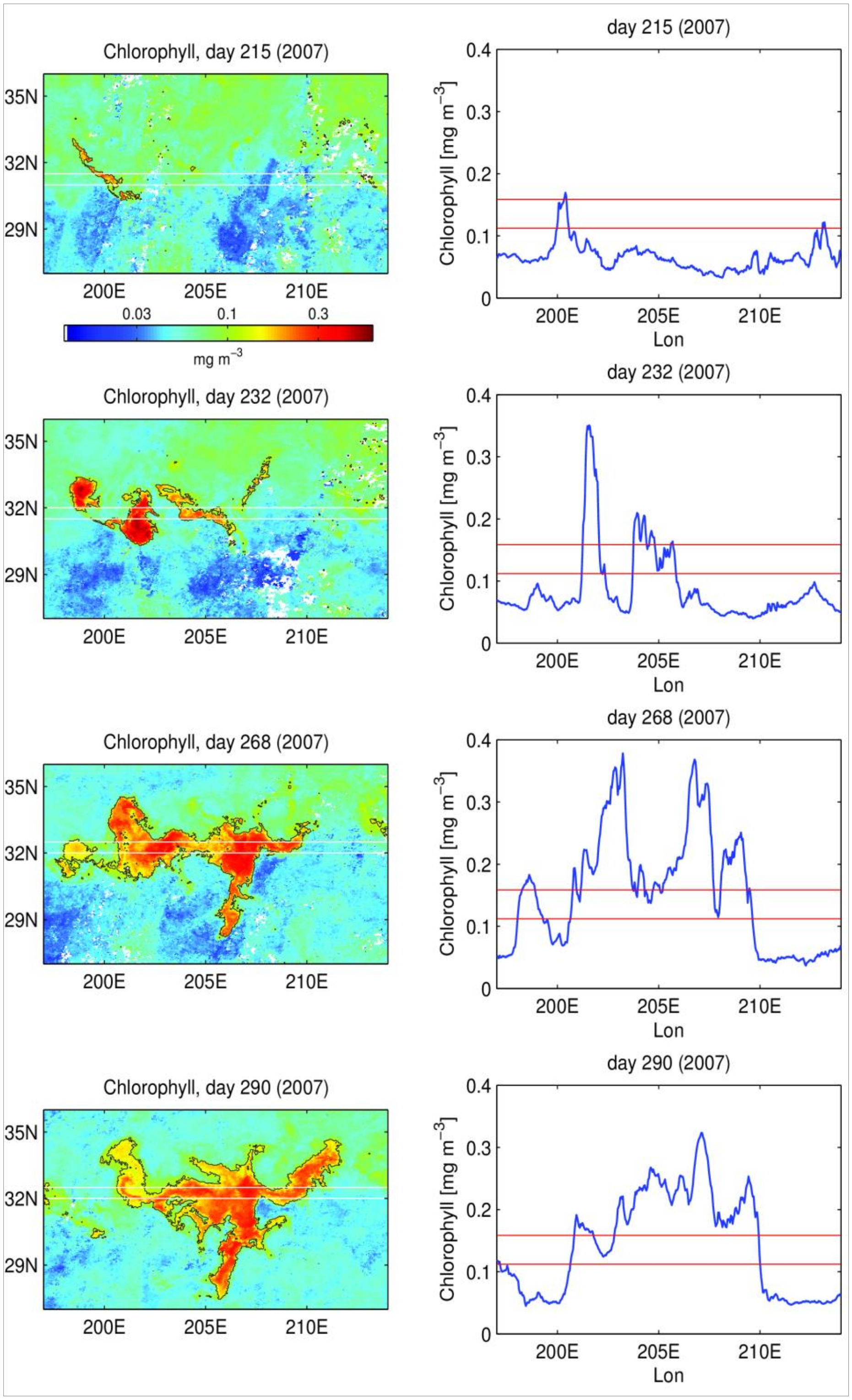

Supplementary Figure 1. Spatio-temporal evolution and patch boundaries of the 2007 bloom. Left panels: Satellite-derived maps of chlorophyll concentrations showing snapshots from the 3 months bloom evolution. Black polygon delineates bloom boundaries as defined by the 0.13 mg m^{-3} chlorophyll contour. delineates the area covered by the cross-section on the right. Right panels: Cross-patch sections of satellite-derived chlorophyll. Red lines mark lowest and highest chlorophyll levels used for delineating patch boundaries (Fig. 2). The figure emphasizes the strong gradients surrounding the patch, which allow unambiguous delineation of its dispersion.

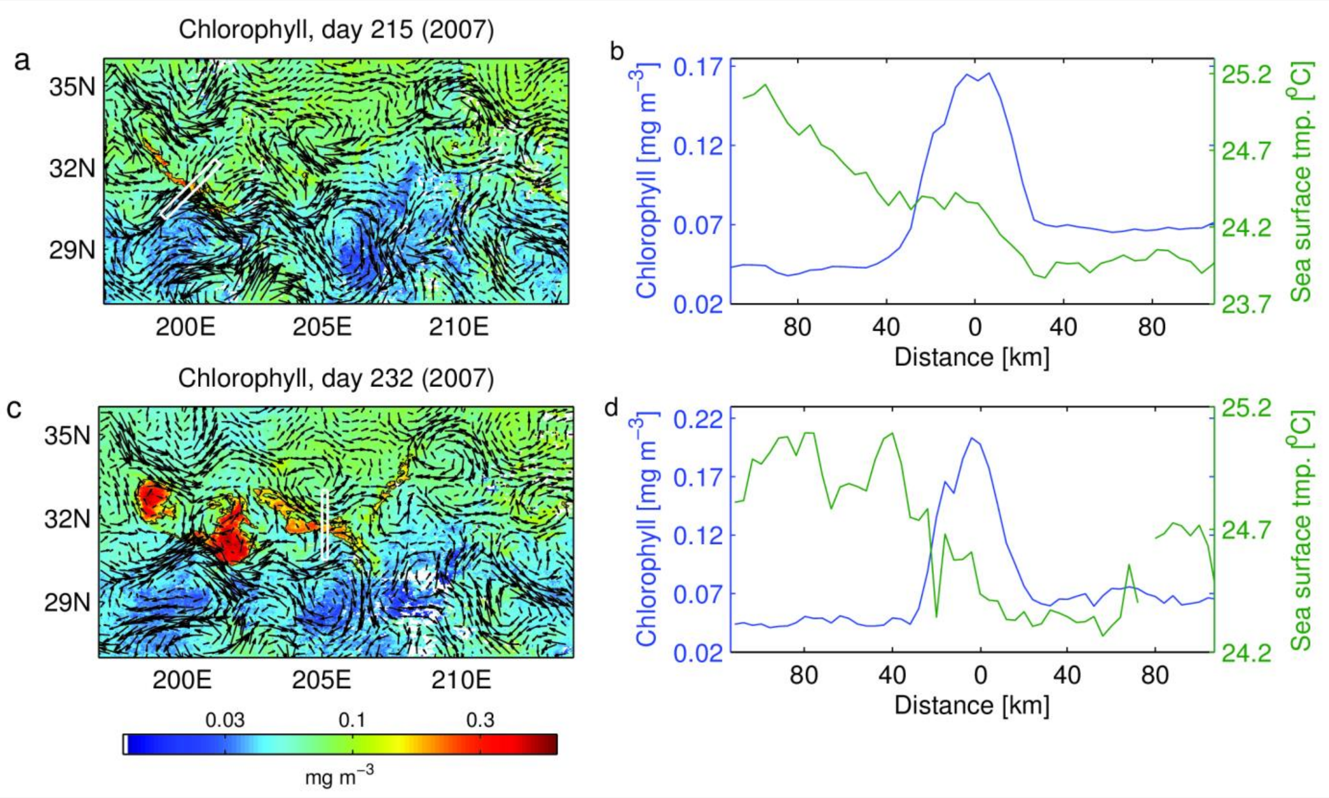

Supplementary Figure 2. Bloom initiation and its association with fine-scale circulation pattern. Left panels: phases of the 2007 bloom as observed in satellite images of surface chlorophyll. Arrows represent velocity vectors of the goestrophic surface currents as derived from satellite altimetry data. White boxes in panels **a** and **c** mark the areas covered in panels **b** and **d**, respectively. Right panels: sections of satellite-derived surface chlorophyll (blue) and temperature (green) across the phytoplankton filaments (black boxes in panels **a** and **c**). Sea surface temperatures were derived from 4 km Level 3 MODIS-Aqua data obtained from the ocean color data distribution site (http://oceandata.sci.gsfc.nasa.gov/)**.** The figures emphasize the co-location of the chlorophyll filaments initiating the bloom and the temperature fronts

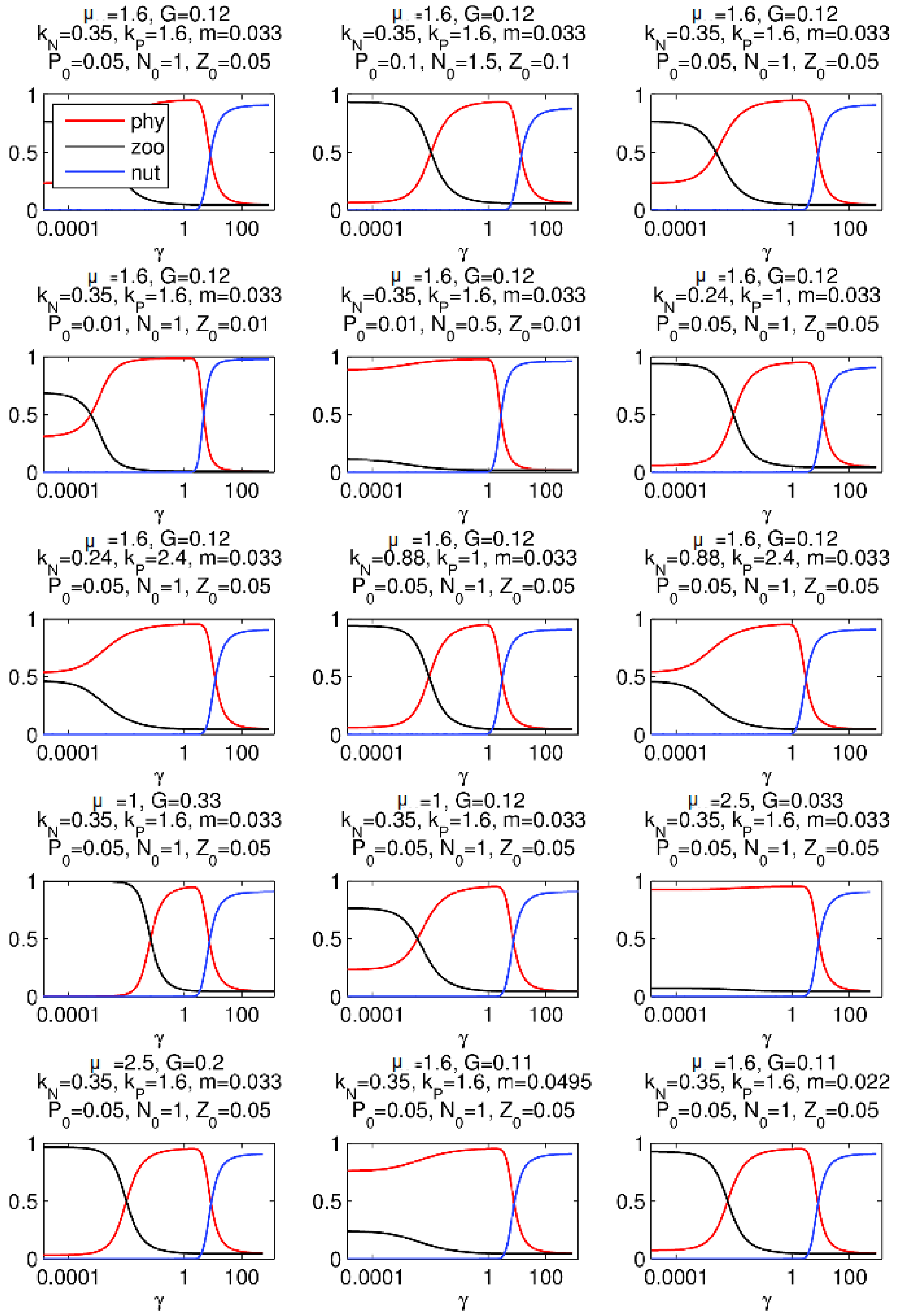

Supplementary Figure 3. **Sensitivity analysis**. Each panel shows diagnostics similar to that presented in Fig. 4a, for model runs with different parameterizations and different initial conditions.

Supplementary Table 1.

Parameters of the ecosystem model.

**Parameter**	**Symbol**	**Value**	**Range**	**Units**
Maximum phytoplankton growth rate	μ	1.6	1 - 2.5	d^{-1}
Grazing rate	G	0.12	0.033 - 0.2	d^{-1}
Nitrate half saturation coefficient	k_N	0.35	0.24 - 0.88	mmol N m^{-3}
Zooplankton mortality rate	m	0.033	0.022 - 0.0495	d^{-1}
Phytoplankton half saturation coefficient	k_P	1.6	1 - 2.4	mmol N m^{-3}
Initial nitrate concentrations	N_0	1	0.5 – 1.5	mmol N m^{-3}
Initial phytoplankton concentrations	P_0	0.05	0.01 – 0.1	mmol N m^{-3}
Initial zooplankton concentrations	Z_0	0.05	0.05 – 0.1	mmol N m^{-3}
Initial volume	V_0	7.5×10^8		m^3
Inlet flow rate	F		1×10^4 -3×10^{12}	m^3 d^{-1}
